# Physical mechanism for biopolymers to aggregate and maintain in non-equilibrium states

**DOI:** 10.1038/s41598-017-03136-7

**Published:** 2017-06-08

**Authors:** Wen-Jong Ma, Chin-Kun Hu

**Affiliations:** 1 Graduate Institute of Applied Physics, National Chengchi University, Taipei, 11605 Taiwan; 2 Institute of Physics, Academia Sinica, Nankang, Taipei, 11529 Taiwan; 3 National Center for Theoretical Sciences, National Tsing Hua University, Hsinchu, 30013 Taiwan; 4 Department of Systems Science, University of Shanghai for Science and Technology, Shanghai, 200093 China

## Abstract

Many human or animal diseases are related to aggregation of proteins. A viable biological organism should maintain in non-equilibrium states. How protein aggregate and why biological organisms can maintain in non-equilibrium states are not well understood. As a first step to understand such complex systems problems, we consider simple model systems containing polymer chains and solvent particles. The strength of the spring to connect two neighboring monomers in a polymer chain is controlled by a parameter *s* with *s* → ∞ for rigid-bond. The strengths of bending and torsion angle dependent interactions are controlled by a parameter *s*_*A*_ with *s*_*A*_ → −∞ corresponding to no bending and torsion angle dependent interactions. We find that for very small *s*_*A*_, polymer chains tend to aggregate spontaneously and the trend is independent of the strength of spring. For strong springs, the speed distribution of monomers in the parallel (along the direction of the spring to connect two neighboring monomers) and perpendicular directions have different effective temperatures and such systems are in non-equilibrium states.

## Introduction

Statistical physics has been applied to understand critical phenomena^1, 2^ of interacting many-body systems via simple model systems very well. In 1952, Yang and Lee^3, 4^ proposed that one can use a lattice gas model, which is equivalent to the Ising model, to describe the liquid-gas critical phenomena. Yang and Lee’s proposal implies that one can use the square well potential for lattice gas particles to represent the Lennard-Jones potential for particles in continuous space. Yang and Lee’s proposal has been confirmed by the results that critical exponents of the three-dimensional (3D) Ising model obtained by Monte Carlo simulations^5, 6^ are consistent very well with experimental measured critical exponents of gas-liquid systems^7^. It has also been found that critical exponents of the three-dimensional Lennard-Jones model system obtained from molecular dynamics (MD) simulations^8^ and Monte Carlo (MC) simulations^9^ are consistent very well with the 3D Ising model^5, 6^, and hence experimental gas-liquid critical systems^7^. In microscopic scales, the Ising model, the Lennard-Jones model system, and experimental gas-liquid critical systems are very different. But they have consistent critical exponents and one can use the Ising model and the Lennard-Jones model system to describe the experimental critical behavior of much complicated gas-liquid systems.

It has been known in the development of molecular biology that many important biological molecules, such as DNA, RNA, peptide, protein, etc, are polymer, called biopolymer. It is interesting to know whether one can use simple model systems of biopolymer to understand interesting biological problems, e.g. folding and aggregation of proteins related to human diseases^10–12^, why a biological system, such as ancient date seeds^13, 14^, can maintain in a viable non-equilibrium state for a very long time^15^, etc.

It has been found that the lattice model of interacting self-avoiding walks^16–21^ can be used to understand the collapse and the freezing transitions of the homopolymer, and a charged H-P model^22, 23^ and multi-state Potts models^24–26^ can be used to understand aggregation of proteins^27^. Lattice models are, however, too simple. In this paper, we use molecular dynamics simulations of polymer chains to understand physical mechanism for biopolymers to aggregate and maintain in non-equilibrium states.

The methods of statistical mechanics have been applied to explain quantitatively very well the energetic origin of the specified conformation for single protein molecule or peptide^10^ as the outcome of searching across all parts of equal probable microscopic states. The non equilibrium processes of biological relevance often, however, probes only very limited parts of those states, which involve collective properties of limited number of molecules under spatial and temporal constrictions. The formation of amyloid-like fibrils from peptides or protein molecules is one of such processes, which is currently in focusing for biomedical researches^11, 22, 28–32^ and for potential material applications^33^. Over the aggregation process, the accumulated domains are composed of lined-up segments of *β*-strands^34^ where the stacking can be enhanced by external confining^35^. The geometric packing, with the assistance of hydrogen-bonding, therefore, plays an important role in the process. Since the efficient stacking of individual protein molecule is essentially guided by the backbone-connectivity^36, 37^, the focus on the latter property and on the combined effects with external confinement may provide the most relevant factors for the processes shared by a wide range of different peptides. Such issues can always be addressed efficiently by studying simplified models^38–40^.

One important issue is on the nature of the process as a nucleation mechanism with its origin from thermodynamic instability. The mechanism is known to be responsible for the ordering of structured chains of homopolymer^41^ and the gelation for stacking of spherical molecules^42, 43^. Some recent experimental results for protein aggregation^44, 45^ are compatible with such an aspect. To focus on those features^35, 44^ shared by the solidification process of simple molecules, in either bulk of small volume^46^ or in confined space^47^, and meanwhile to underscore the effects of backbone connectivity in a systematic manner, we have initiated studies by molecular dynamics simulations^48, 49^, on the aggregation problem of dilute polymer chains with effective soft confinement imposed by the finite space and the boundary conditions in simulation. While the main bodies of the clusters formed in these simulations are in bundled structures, which are different from the ordered layers obtained in processes of crystallization in polymer melt^50^, the dynamics of ordering of the dilute chains can still be quantified effectively^48^. It is also shown^48, 49^ that we can manipulate the ruggedness along the backbones to prepare systems of polymer chains which intrinsically do not undergo processes of aggregation. For these systems, enhanced stiffness may render the chains stay at non-equilibrium ‘quasi-steady’ situations, which show unusual thermal equilibration properties in presence of contact with background fluid and have non Maxwell-Boltzmann velocity distributions for the monomers^51, 52^. One question of particular interest is then whether this kind of stability implicitly maintained by the backbone connectivity is also present in the middle of aggregation processes occurring for those systems which are subject to thermodynamic instability.

**Figure 1:**
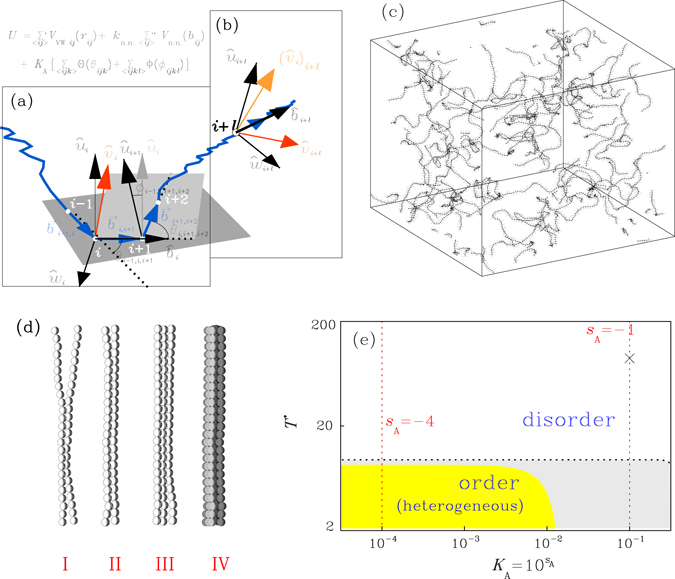
Model system of *N*_*P*_ = 40 nearly-homogeneous polymer chains mixed with vapor of *N*_*F*_ = 6000 spherical atoms; each chain has *n* = 100 monomers including a tiny amount (5%) randomly placed impurities with different interactions; the monomers interact via potential *U*, controlled by strength parameters ${k}_{{\rm{n}}.{\rm{n}}.}\equiv {10}^{s}$, for nearest-neighbor-bonding, and ${K}_{{\rm{A}}}\equiv {10}^{{s}_{{\rm{A}}}}$, for angle potential (see *Methods* for details): (**a**) The coordinate system with basis vectors {$\hat{b}$, $\hat{u}$, $\hat{w}$} and bending and torsion angles, (*θ*, $\varphi$) for the curved backbone of one chain; (**b**) the inner product ${({\hat{v}}_{i})}_{i+l}\cdot {\hat{v}}_{i+l}$ defined between the directions of motions of two monomers at positions *i* and *i* + *l*, in reference to the curved coordinates; (**c**) a snapshot of backbone coordinates from simulation for a system with the angle potential strength *K*_A_ = 10^−1^; (**d**) stages of aggregation: stage I for local lining-ups between segments of two chains, stage II for longitudinal extensions of ordered segments along the chains, stages III and IV for transverse growths to have belt-like two-dimensional local orderings and bundle-like three-dimensional structures, respectively; (**e**) schematic diagram (in a log-log plot) for equilibrium states (in white color) and steady phases subject to clustering (in grey or yellow color), under the specifically selected setup on the potential *U* (see *Methods*). The diagram in plot (e) covers a range of two decades in temperature and a range more than three decades in *K*_A_. In simulation, the stages I to IV for clustering are quantitatively identified by the changes in the values of parameters, *R*_0_, *R*_1_ and *R*_2_, respectively, over time (see *Methods*). In Fig. 6, we show the simulation data for systems evolving in time along the red dashed line on the left side of plot (e). In the colored region below the dashed black line, the systems are subject to thermodynamic instability, which drives the initially random and uniform configurations of chains to accumulate. Only those in the yellow region can extend the ordered segments along the backbones (stage II) sufficiently to trigger the formation of transverse domains (stage III) and succeed in aggregation to reach the stage IV with bundled structures in major clusters^48, 49^. For the clarity of presentation, the beads shown in the schematic plot (d) do not reflect the sizes of the monomers. The diameter *σ* (see *Methods*) of the monomers used in simulations is about three times of the bond length (see *Methods*). In simulation, the assignment *s*_A_ = −∞ leads to the cases (not shown in plot (e)) in which we turn off the angle potentials. We also allow to have *s* = ∞, in which cases the bonds are rigid in fixed lengths. In general, the locations of boundaries between the three regions of plot (e) (in white, grey and yellow colors, respectively) are not sensitive to the strength parameter *k*_n.n._. The snapshot in plot (c) has *s* = 4 and is at a state marked by ‘x’ in plot (e).

The model systems under our consideration are in fixed volume, with their steady non equilibrium phases within the thermodynamically unstable regime (Fig. 1) determined by the strength parameter *K*_A_ of angle potential along the chains in powers of ten, ${K}_{{\rm{A}}}={10}^{{s}_{{\rm{A}}}}$. The collection of chains will have a bundled structure after going through the process from stage I to stage IV (Fig. 1(d)), reaching steady phases in the lower temperature regime of Fig. 1(e), if *K*_A_ of the system is sufficiently small (in yellow colored region). We have chosen a specific minimum for the bending angle potential (see *Methods*) to introduce local hindrance into individual chains, which prevents the backbones from forming lined-up segments and the ordering is prohibited as soon as the strength *K*_A_ is increased to push the system entering the grey colored region in Fig. 1(e). Under the circumstance the stage IV can never be reached and the system is left with configurations of partially ordered local conformations. The occurrence of ordering is insensitive to the bonding strength *k*_n.n._ and is enhanced but not determined by the spatial confinement^48, 49^.

In previous studies^51, 52^, we have revealed in simulations, the primitive form of backbone induced stability for systems having *s*_A_ = −1, which undergo no clustering at all temperatures (Fig. 1(e)). The simulations have been carried out *without* imposing external temperature controls and the numerical noises in the simulations are treated as unavoidable sources of heat, causing the systems out of equilibrium, with temperatures varied in time (along the red dashed line on the right of Fig. 1(e)). The backbone-induced stability of the system displays itself in form of a temperature-reduced velocity distribution. In contrast to the clustering property, the distribution is determined by the parameter *k*_n.n._ in powers of ten, *k*_n.n._ = 10^s^. Such a property has not been examined systematically, however, for systems which undergo aggregation^48, 49^, partly because the latter processes are often carried out under external thermal controls^49^. In this study, we look into the underlying origin of the stability previously found for those systems with *s*_A_ = −1 and then extend the analysis to systems with *s*_A_ = −4 over the range of temperatures marked by red dashed line on the left of Fig. 1(e), where the systems undergo processes of aggregation. All simulations are carried out with no controls of temperature.

In conjunction with a macroscopic scenario, the time evolution of a system in molecular dynamics (MD) simulation, consists of a series of step-by-step small changes involve the change *δ* **U** in internal energy, the macroscopic work *δ* **W** done by the system and the adsorbed heat *δ* **Q** at a macroscopic level, in terms of the equality (of the first law)

1 $$\delta {\bf{U}}=\delta {\bf{Q}}-\delta {\bf{W}}.$$

In contrast to the simulation of a fixed equilibrium state, in which the macroscopic work^53, 54^ and the heat^54, 55^ are often controlled externally to make those step-by-step changes fluctuating in the *vicinity* of one single macroscopic state, the series of changes over a non equilibrium relaxation process involves states which may have fundamentally different spatial symmetry or heterogeneity. In MD simulation of equilibrium states, the external controls are based on equilibrium statistical mechanical schemes^53–56^, designed to generate well defined probability functions^54^, often with controlling designed in accord with the structural features^53, 57, 58^. In simulating non equilibrium processes with time-varying symmetry or heterogeneity, it is necessary to be cautious in using any external control and better to allow the probability functions be determined by underlying micromechanics, when the interested phenomena, such as the clustering of polymer chains caused by thermodynamic instability, are over limited characteristic length and time scales. The dynamic features dominated by the backbones can be totally preserved if the simulations be carried out without imposing external controls. Though such a strategy has the drawback that the heat *δ* **Q** contributed by numerical noises is not controlled and the temperature of the system is floating, we do in our studies^51, 52^ revealed the stability implicitly supported by the backbone connectivity.

In contrast to the schemes of Monte Carlo simulation^59, 60^, where one considers the probability function of microscopic states as a function of total potential energy and temperature *T* is used as a control parameter, the micromechanics-based schemes of MD simulations allow to measure *T* by realizing it as a kinetic parameter, determined via the probability density function *G*(**K**_1_) of the *thermal* kinetic energy **K**_1_ of one particle. The realization can be effectively applied in considering a non equilibrium relaxation process, in which *G* is a time varying function, specifying the microscopic state of the system at any instant. It is not difficult to realize that the thermodynamic concept of ‘quasi-static process’ justifies using one single probability function $\tilde{G}$ for a slow changing non equilibrium process composed of a series of nearly equilibrium states with different values in *T*, as soon as the kinetic energy being scaled by *T*.

For an equilibrium state, satisfying Maxwell-Boltzmann statistics, the scaled function $\tilde{G}$ reads

2 $$\tilde{G}({\tilde{{\bf{K}}}}_{1})=J({\tilde{{\bf{K}}}}_{1}){e}^{-{\tilde{{\bf{K}}}}_{1}},$$

where the ${\tilde{{\bf{K}}}}_{1}=\beta {{\bf{K}}}_{1}$ is the scaled variable for single-particle kinetic energy **K**_1_, times the energy scale factor $\beta =\frac{1}{{k}_{B}T}$ with the mean kinetic energy $\langle {{\bf{K}}}_{1}\rangle =\frac{\nu }{2}{k}_{B}T$ for *ν* degrees of freedom (*k*_*B*_: the Boltzmann constant) and $J({\tilde{{\bf{K}}}}_{1})=B\sqrt{{\tilde{{\bf{K}}}}_{1}}$ is the Jacobian factor with normalization constant *B*. We emphasize that the property holds for the thermal part of kinetic energy, on the basis of an *isotropic* distribution in directions of motions. In a flow, for example, **K**_1_ is contributed by the particle velocity with the local flow velocity being subtracted off.

In this study, we explored the possibility of an extension of such a scheme, to include the situations where backbones dominate the motions of the monomers, in ‘softly confined’ systems of stiff polymer chains. In these systems, the restricted size and limited time span of the interested phenomena prevent the system from being effectively comprehended by equilibrium statistical mechanics. Instead, from the results of MD simulations for systems with *s*_A_ = −1^51, 52^, where no external controls are imposed, we have seen some evidences that, the presence of backbone-supported dynamic stability distinguishes the states of reaching ‘quasi-steady’ situations from those staying transient. We have an extended form for the scaled function Eq. (2),

3 $$\tilde{G}({\tilde{{\bf{K}}}}_{P,1})={J}_{q}({\tilde{{\bf{K}}}}_{P\mathrm{,1}}){e}_{q}^{-{\tilde{{\bf{K}}}}_{P\mathrm{,1}}},$$

with ${J}_{q}({\tilde{{\bf{K}}}}_{P\mathrm{,1}})={B}_{q}\sqrt{{\tilde{{\bf{K}}}}_{P\mathrm{,1}}}$ and the exponential function *e* ^*x*^ in Eq. (2) replaced by the generalized exponential function ${e}_{q}^{x}$, defined by ${e}_{q}^{x}\equiv {\mathrm{(1}+\mathrm{(1}-q)x)}^{\frac{1}{1-q}}$ ^61, 62^. *q* ranges $1\le q\le \frac{7}{5}$ and the case *q* = 1 resumes the ordinary exponential function. In a ‘quasi-steady’ situation, the values of *q* for all instants fall in a narrow range around a fixed value q*. It highlights the notion that those states are considered as an integrated situation at non equilibrium.

To be specific, we consider the processes of thermal equilibration between polymer chains and background fluid in six systems with the angle potential by the parameter *s*_A_ = −1 and with, respectively, the parameter for bonding strengths *s* = 0, 1, 2, 3, 4^51^ and ∞ ref. 52. Over the processes along the red dashed line on the right side of Fig. 1(e), the probability density function *P*_GMB_(*v*) for monomer speed *v* at each instant is well fitted by the generalized Maxwell-Boltzmann (GMB) speed distribution

4 $${P}_{{\rm{GMB}}}(v)={A}_{q}{\beta }^{3/2}{j}_{G}(v){e}_{q}^{-\beta \tfrac{m{v}^{2}}{2}},$$

where the factor *j*_*G*_(*v*) = 4*πv* ^2^ has been used in previous studies^51, 52^ and *A*_*q*_ is the normalization factor and all monomers are assumed to have equal masses, *m* = 1. Note that, the choice of factor 4*πv* ^2^ is compatible with the isotropic Maxwell’s distribution of speed (subscript ‘IMB’ for istropic Maxwell-Botzmann), which is

5 $${P}_{{\rm{IMB}}}(v)={(\frac{m}{2\pi {k}_{B}T})}^{\frac{3}{2}}4\pi {v}^{2}{e}^{-\frac{m{v}^{2}}{2{k}_{B}T}},$$

as the special case of *q* = 1. In this case, the temperature *T* is determined by ${k}_{B}T=\frac{\langle m{v}^{2}\rangle }{\nu }$. Specifically, we have the number of degrees of freedom *ν* = 3, for systems of finite *s*. The factor *j*_*G*_(*v*) will be discussed further in the last paragraph of Discussion. The “quasi-steady” refers to the situations that the fitted values of *q* for all instants of a period of time fall over a narrow range measured by ±Δ*q* around a fixed value q*. The distributions ${\tilde{P}}_{{\rm{GMB}}}(\tilde{v})$ for the scaled speed $\tilde{v}={\beta }^{\mathrm{1/2}}v$ for all instants are then well approximated by one single master curve given by refs 51, 52

6 $${\tilde{P}}_{{\rm{GMB}}}(\tilde{v})={A}_{{q}^{\ast }}4\pi {\tilde{v}}^{2}{e}_{{q}^{\ast }}^{-\frac{{\tilde{v}}^{2}}{2}}.$$

Correspondingly, the scaled function $\tilde{G}({\tilde{{\bf{K}}}}_{P\mathrm{,1}})$ for scaled monomer kinetic energy ${\tilde{{\bf{K}}}}_{P\mathrm{,1}}=\beta {{\bf{K}}}_{P\mathrm{,1}}$ reads $\tilde{G}({\tilde{{\bf{K}}}}_{P\mathrm{,1}})={\tilde{P}}_{{\rm{GMB}}}(\sqrt{2{\tilde{{\bf{K}}}}_{P\mathrm{,1}}/m})/\sqrt{2m{\tilde{{\bf{K}}}}_{P\mathrm{,1}}}$.

Each of the six systems has *N*_*P*_ = 40 chains of *n* = 100 monomers in length, mixed with a background vapor of *N*_*F*_ = 6000 spherical atoms. All of these systems had evolved in time from initial microstates of the same quenched configurations and undergo relaxations toward thermal equilibration between polymer and fluid, which is signaled by the reaching of constancy in the ratio

7 $${\rm{\Gamma }}=\frac{{{\bf{K}}}_{P\mathrm{,1}}{\nu }_{F}}{{{\bf{K}}}_{F,1}{\nu }_{P}},$$

where we put *ν*_*P*_ = 3 and *ν*_*F*_ = 3, to underscore the numbers of degrees of freedom of the monomer and the fluid atom, respectively. In equilibration, Γ is allowed to reach a value ranging between $\frac{201}{300}$ and 1. The lower bound $\frac{201}{300}$ is for the cases of rigid bonding (*s* = ∞), where 99 bond constraints are present for a chain composed of *n* = 100 monomers. While the evaluation does not take the collective modes as the basis in counting the numbers of degrees of freedom, the constancy in Γ does behave as a convenient signature to identify the reaching of the peculiar equilibration between the two parts, in spite of the varying temperatures for both parts over time. The thermal equilibration *within* the collection of polymer chains in the mixture, on the other hand, is signaled by the afore-mentioned scaling in the distribution of monomer velocities (Eq. (6)). Specifically, the fitted *q*’s are in a range estimated by q* ± Δ*q*, which are 1.032 ± 0.019 for *s* = 0, 1.025 ± 0.017 for *s* = 1, 1.028 ± 0.020 for *s* = 2, 1.075 ± 0.026 for *s* = 3, 1.140 ± 0.026 for *s* = 4 and 1.169 ± 0.047 for *s* = ∞, respectively. They show an increasing trend with *s*. Those averages are taken on snapshots over time periods of 30–40*τ*, in intervals of 0.25*τ* (see *Methods*, for the definition of time unit *τ*).

## Results

**Figure 2:**
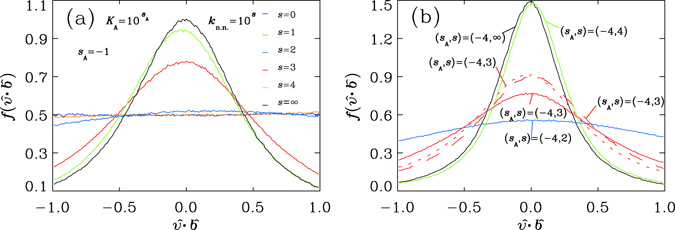
Distribution functions *f* for the direction cosine $\hat{v}\cdot \hat{b}$ between the velocity direction $\hat{v}$ of a monomer and its bond direction $\hat{b}$ along a polymer chain in systems described in Fig. 1: (**a**) with the same strength of angle potential, *s*_A_ = −1 (dashed red vertical line on the right side of Fig. 1(e)) and with different stiffness *s* = 0, 1, 2, 3, 4^51^ and ∞ ref. 52, respectively, undergoing no aggregation; (**b**) with (*s*_A_, *s*) = (−4, 2), (−4, 3), (−4, 4) (﻿in this study,﻿ dashed red vertical line on the left side of Fig. 1(e)) and (−4, ∞)^48^, which undergo aggregation processes without external controlling on temperatures. Each of the distributions is obtained by collecting data of 2000 snapshots, in intervals of 5 steps, over a quasi-steady situation. of period 0.25*τ* in time step *δt* = 2.5 × 10^−5^ *τ* (see *Methods*). In plot (b), we include three curves for the distributions of the system with (*s*_A_, *s*) = (−4, 3) (collected over time intervals at *t* = 293.5 (red dashed line), *t* = 353.5 (red broken line) and *t* = 822.5 (red solid line), respectively, in Fig. 6). The corresponding values for *q* can be found in Fig. 4 (for plot (a)) or Fig. 5 (for plots (a,b))

Figure 2(a) shows how the backbones affect the motion of monomers, in the distribution of the cosine $\hat{v}\cdot \hat{b}$ between the direction $\hat{v}=\overrightarrow{v}/|\overrightarrow{v}|$ of velocity $\overrightarrow{v}$ for a monomer and the tangent direction $\hat{b}=\overrightarrow{b}/|\overrightarrow{b}|$ along the backbone with bond vector $\overrightarrow{b}$ at that monomer site (Fig. 1(a)), for those six systems with *s*_*A*_ = −1. The enhanced probability emerges in the central part of the distribution when the stiffness increases from *s* = 0 to *s* = ∞, indicating the trend in dividing kinetic energy with respect to the backbone coordinates, with a preference in the transverse direction. One important feature of the distribution function $f(\hat{v}\cdot \hat{b})$ is the symmetry between $\hat{v}\cdot \hat{b}$ and −$\hat{v}\cdot \hat{b}$, as the result of the governance of backbones. It suggests the curved coordinates along the backbone (Fig. 1(a)) be the proper reference to quantify the motion. Figure 2(b) shows the distribution of the cosine $\hat{v}\cdot \hat{b}$ for systems of fixed *s*_*A*_ = −4 and *s* = 2, 3, 4, and ∞, respectively. Again, as *s* increases, the distribution has larger values near $\hat{v}\cdot \hat{b}=0$.

**Figure 3:**
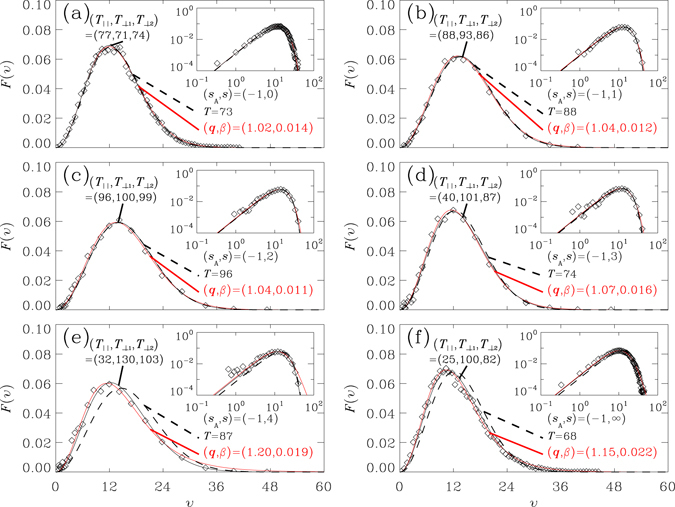
Speed distribution functions *P*(*v*) as functions of speed *v* for monomers and their fittings. The function is computed in such a way that the area ${\int }_{0}^{\infty }\,P(v)dv$ under each curve is unity. Plots (a–f) are, correspondingly, for the six systems considered in Fig. 2(a). Simulation data^51, 52^ (open circles) are the results averaged over 40 snapshots, collected over a period 0.002*τ* (see *Methods*). In each plot, the simulation data are compared with the fitted curves of the generalized Maxwell-Boltzmann (GMB) speed distribution *P*_GMB_(*v*) defined in Eq. (4) (red solid line), of isotropic Maxwell-Boltzmann (IMB) function Eq. (5), having the same second moment 〈*v* ^2^〉 as simulation data (dashed line) and of *P*_EMB_(*v*), the elliptic Maxwell-Boltzmann (EMB) defined in Eq. (8) (black solid line). While all three fittings provide approximately the same curve as the simulation data for plots (a–c), both the fittings by GMB and by EMB agree with the simulation data better than it is for IMB, for plots (d–f). Note that the values for *q* shown in the plots are obtained by the fittings over 0.002*τ* and fall in the ranges q* ± Δ*q* (listed in the text below Eq. (7)) obtained by averaging over time periods of 30–40*τ*.

One immediate refinement of the information on the anisotropy in motion in reference to the backbone coordinates is to decompose the velocities of individual monomers into the tangential and the transverse components and fit the distributions approximately by one-dimension Maxwell-Boltzmann functions. While there are recognizable deviations between the data and the fitted curves, for those cases with strong anisotropy in motion, such as the systems with *s* = 4 and *s* = ∞, in treating them as if they were statistically mutually independent components, the combination into an elliptical Gaussian distribution^63^ does provide an acceptable approximation for velocity distribution in three-dimension. Figure 3 shows such fittings for the six systems with *s*_*A*_ = −1 by using the ‘elliptical Maxwell-Boltzmann’ (EMB) distribution, defined by

8 $${P}_{{\rm{EMB}}}(v)={A}_{{\rm{E}}}{j}_{{\rm{E}}}(v){e}^{-\frac{m{v}^{2}}{2{k}_{B}{T}_{\perp }}}$$

where the Jacobian factor

$${j}_{{\rm{E}}}(v)={\mathrm{(2}\pi )}^{\frac{3}{2}}{(\frac{{k}_{B}}{m})}^{\frac{1}{2}}{(\frac{{T}_{\parallel }{T}_{\perp }}{{T}_{\perp }-{T}_{\parallel }})}^{\mathrm{1/2}}\times v\times erf(v{\{\frac{m}{2{k}_{B}}[\frac{1}{{T}_{\parallel }}-\frac{1}{{T}_{\perp }}]\}}^{\frac{1}{2}}),$$

and the normalization constant

$${A}_{{\rm{E}}}={(\frac{m}{2\pi {k}_{B}})}^{\frac{3}{2}}{({T}_{\parallel }{T}_{\perp }^{2})}^{-1/2}.$$

The error function *erf*(.) is defined by ${\rm{e}}{\rm{r}}{\rm{f}}(x)=\frac{2}{\sqrt{\pi }}{\int }_{0}^{x}\,{e}^{-{t}^{2}}dt$ and the transverse motion is described by one parameter ${T}_{\perp }=\frac{1}{2}({T}_{\perp 1}+{T}_{\perp 2})$, with ${T}_{\parallel }$, ${T}_{\perp 1}$ and ${T}_{\perp 2}$ obtained by fitting the components of monomer velocities in parallel (marked by subscript ‘‖’) and in two perpendicular directions (marked by subscripts ‘⊥1’ and ‘⊥2’, respectively) in reference to the curved backbone coordinates, corresponding to $\hat{b}$, $\hat{u}$ and $\hat{w}$, respectively (Fig. 1(a)). In the limit ${T}_{\perp }\to {T}_{\parallel }$, we have *j*_E_(*v*) → 4*πv* ^2^ and Eq. (8) converges to the isotropic case, Eq. (5). The values listed for the fitted curves in Fig. 3 show that, for those systems with $f(\hat{v}\cdot \hat{b})$ (Fig. 2(a)) deviating from the uniform distribution, their *T*_⊥1_ and *T*_⊥2_ are significantly larger than *T*_‖_ (Fig. 3(d–f)). The polymer chains, under the dominance of backbones, are internally in *non*-*equilibrium* situations.

The fitting by EMB (Eq. (8)) is certainly better than that by the isotropic Maxwell-Boltzmann distribution (IMB) (Eq. (5)) and is comparable with the result by Eq. (4). Equation (8) suggests a scenario of thermal motion in a ‘tube’-like string^37, 64–66^. Note that, while both Eqs (4) and (8) are two-parameter fitting functions, the analysis using Eq. (4) does not require the knowledge of backbone frames and provide a convenient way to reveal the stability of the system, by simply fitting to obtain a scaled distribution function covering all instants of time. Besides, the validity of expression Eq. (4) ^67^ does not rely on the statistical independence among the components of velocity, along the longitudinal and in the transverse directions. In previous studies^51, 52^, it has been shown that the fitting by Eq. (4) and, thereof, by Eq. (6) for sequences of microscopic states, do identify effectively the non-equilibrium ‘quasi-steady’ states, generated by untamed numerical noises. In this study, we retained the same factor *j*_*G*_(*v*) = 4*πv* ^2^, and found Eq. (4) still effective for analyzing non-equilibrium situations, originated from thermodynamic instability.

Our next task is then to extend the temperature-reduced approach to resolve the following issues. Firstly, while the parameters Γ and *q* provides convenient signatures to find, respectively, the external and internal thermal equilibrations for a collection of polymer chains, we are lacking of knowledge on the underlying origins of the stability leading to the equilibration. Are they from the same origin or not? Secondly, the equilibration reached in six systems considered so far are derived from the same initial preparation. Over longer time scales for sufficient relaxations, allowing to erase the residual memory of initially excited collective modes^68^, in what form will the equipartition of energy prevail in these systems? Thirdly, the non-equilibrium situations considered so far are in forms of temperature varying caused by external source (heat from numerical noises). Whether and how the governance by backbone could sustain the stability under the intrinsic changes in a process of aggregation is subject to be examined. Fourthly, it is not clear whether there is a system in the class of model chains under our consideration *does not* have a backbone dominant ‘quasi-steady’ stability?

We proceed to analyze the internal correlations along the chains, to underscore the effects of backbone connectivity. The quantities of which the pair correlation to be analyzed should not be directly dependent on temperature and should reflect the orientations of local coordinates of the curved frame along the backbones.

### Correlation-anti-Correlation Symmetry in directions of motions

**Figure 4:**
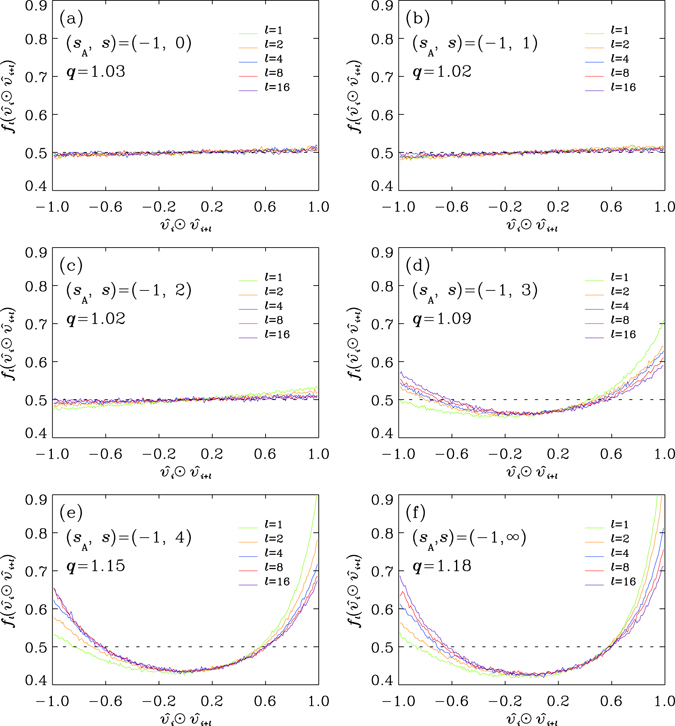
Typical distributions *f*_*l*_ of the cosine ${\hat{v}}_{i}\odot {\hat{v}}_{i+l}$ = ${({\hat{v}}_{i})}_{i+l}\cdot {\hat{v}}_{i+l}$ = ${\hat{v}}_{i}\cdot {({\hat{v}}_{i+l})}_{i}$ (Fig. 1(b)) for *l* = 1, 2, 4, 8 and 16, observed in the curved coordinates, for mixture systems described in Fig. 2(a), with stiffness indices (**a**) *s* = 0, (**b**) *s* = 1, (**c**) *s* = 2, (**d**) *s* = 3, (**e**) *s* = 4 and (**f**) *s* = ∞, respectively. Data are averaged over 10000 snapshots, collected over a period 0.25*τ* (see *Methods*). The dashed horizontal line marks the uniform distribution ${f}_{{\rm{UI}}}({\hat{v}}_{i}\odot {\hat{v}}_{i+l})=0.5$ for the fully isotropic situation. We also list the values for the corresponding *q*-parameter obtained by the fittings of the generalized Maxwell-Boltzmann (GMB) speed distributions (Eq. (4)).

We consider the pair correlation between the directions of motion for monomers along a chain, measured by the probability density *f*_*l*_ of direction cosine ${({\hat{v}}_{i})}_{i+l}\cdot {\hat{v}}_{i+l}$ (or, equivalently, ${\hat{v}}_{i}\cdot {({\hat{v}}_{i+l})}_{i}$) observed along the curved coordinates, where we record the relationship between ${\hat{v}}_{i}$ and the local orientations at site *i* (Fig. 1(a)) and revive ${\hat{v}}_{i}$ by resuming the relationship in the new local coordinates at site *i* + *l*, as ${({\hat{v}}_{i})}_{i+l}$ (Fig. 1(b)). We briefly denote the inner product ${({\hat{v}}_{i})}_{i+l}\cdot {\hat{v}}_{i+l}$ and, equivalently, ${\hat{v}}_{i}\cdot {({\hat{v}}_{i+l})}_{i}$ as ${\hat{v}}_{i}\odot {\hat{v}}_{i+l}$. Figure 4 shows the typical curves of *f*_*l*_’s, for *l* = 1, 2, 4, 8 and 16, computed over time intervals of the same duration as that considered in Fig. 2(a), which is 125 times of the period employed to compute the speed distributions in Fig. 3. The distributions for smaller *l’s*, in all of the six systems, are highly asymmetric between the positive and the negative sides of ${\hat{v}}_{i}\odot {\hat{v}}_{i+l}$. The escalated probability density around ${\hat{v}}_{i}\odot {\hat{v}}_{i+l}=1$. indicates the tendency of being parallel in motion for a pair of near neighbors. The tendency is dominantly stronger for the three cases with stronger bonds (Fig. 4(d–f)), in comparing with the other three (Fig. 4(a–c)), where *f*_*l*_’s are close to be uniform for all *l*’s. The deviation from a uniform distribution increases with the enhanced bonding (by increasing *s*). For larger *l*’s, the asymmetry fades away, that we see the emergence of symmetrized *f*_*l*_’s, with equal probability to be parallel-bound with a value ${\hat{v}}_{i}\odot {\hat{v}}_{i+l}=x( > \mathrm{0)}$ and its anti-parallel counterpart with ${\hat{v}}_{i}\odot {\hat{v}}_{i+l}=-x( < \mathrm{0)}$, in directions of motion between a pair of monomers separated by *l* bonds along a chain. Up to *l* = 16, both the convergence in sequences of *f*_*l*_’s, measured by the difference between *f*_*l*_ and *f*_*l*+1_, and the asymmetry between ${\hat{v}}_{i}\odot {\hat{v}}_{i+l} > 0$ and ${\hat{v}}_{i}\odot {\hat{v}}_{i+l} < 0$, estimated by the difference between *f*_*l*_(*x*) and *f*_*l*_(−*x*), are comparable with the error bars of the data.

**Figure 5:**
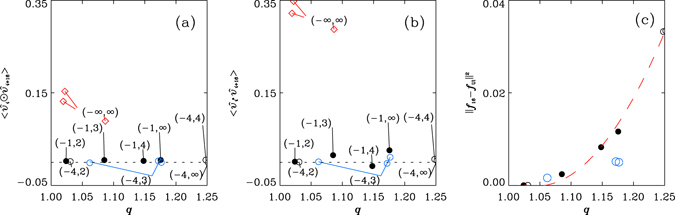
(**a**) The two-dimensional (2D) plot of the mean $\langle {\hat{v}}_{i}\odot {\hat{v}}_{i+16}\rangle ={\int }_{-1}^{1}\,{f}_{16}(x)xdx$ (Fig. 4) and *q* for systems with their parameters (*s*_A_, *s*) marked in the plot, the inner product is evaluated in terms of backbone coordinates, defined in Fig. 1(b); (**b**) the 2D plot of the mean $\langle {\hat{v}}_{i}\cdot {\hat{v}}_{i+16}\rangle$ evaluated in terms of simulation-box (Fig. 1(c)) coordinates and *q*; (**c**) the normed square deviation ${\Vert {f}_{16}-{f}_{{\rm{UI}}}\Vert }^{2}$ defined by Eq. (10) as functions of *q*, for the six systems (filled black circle) with *s*_A_ = −1 and *s* = 2, 3, 4 and ∞, respectively, obtained with the data of *f*_*l*_, *l* = 16 plotted Fig. 4. These systems do not have aggregation processes. We also include the results for *s*_A_ = −4, and *s* = 2, 4 and ∞, respectively, each of which has one data (in open black circle) in each of (**a**–**c**). There are three data points for (*s*_A_, *s*) = (−4, 3) (in open blue circle) in each figure, which have different values in *q* (at time *t* = 293.5, *t* = 353.5 and *t* = 822.5, respectively, in Fig. 6(b)). These four systems with *s*_A_ = −4 undergo aggregation processes. The data for those eight systems follow the same trends in plot (c), with eye-guidance by the dashed lines, except for the three data points for the system with (*s*_A_, *s*) = (−4, 3). In (**a**), the values of $\langle {\hat{v}}_{i}\odot {\hat{v}}_{i+16}\rangle$ are all vanishingly small for all the eight systems mentioned above (denoted by filled or open circles in black or blue colors), as the results of the correlation-anti-correlation symmetry, that between ${f}_{16}({\hat{v}}_{i}\odot {\hat{v}}_{i+16})$ and ${f}_{16}(-{\hat{v}}_{i}\odot {\hat{v}}_{i+16})$. The data points in plot (b) are systematically larger in magnitudes than those of their counterparts in plot (a), suggesting the unique status of the backbones in referring to the symmetry, except the not-so-obvious results for the two cases with the weakest stiffness in each category, (*s*_A_, *s*) = (−1, 2) and (*s*_A_, *s*) = (−4, 2) and the two cases with strong stiffness in the category of *s*_A_ = −4, that is, (*s*_A_, *s*) = (−4, 4) and (*s*_A_, *s*) = (−4, ∞. The analysis on the values of skewness of the distributions *f*_*l*_’s for the cases of strong stiffness (*s*_A_, *s*) = (−4, 4) and (*s*_A_, *s*) = (−4, ∞), nevertheless, do show the consistent trend that support the above-mentioned conjecture. The directions of motion in all these eight systems are, therefore, in backbone-constrained isotropy, including the system with (*s*_A_, *s*) = (−4, 3), even though the latter has varying q* over time (Fig. 6(b)). We also include, in plots (**a**,**b**), three data points (red diamonds) for a system with (*s*_A_, *s*) = (−∞, ∞) (i.e. with rigid bond and vanishing angle potentials) and in absence of background fluid^48^. In this case, *q* varies over time in the aggregation process. The values in $\langle {\hat{v}}_{i}\odot {\hat{v}}_{i+16}\rangle$ for this system are much larger than those for the other eight systems and are not vanishingly small. The direction of motion is not in backbone-constrained isotropy in this case. Note that, the two systems with the weakest stiffness considered in the plots, (*s* = 2, those for (*s*_A_, *s*) = (−4, −2) and (*s*_A_, *s*) = (−1, 2) have their normed square deviation ${\Vert {f}_{16}-{f}_{{\rm{UI}}}\Vert }^{2}$ close to zero. Each of the two systems show equally well correlation-anti-correlation symmetry, with the inner product computed in either backbone coordinates (plot (a)) or simulation-box coordinates (plot (b)), because of the uniform isotropy in *f*_16_.

Such a symmetry in the probability density function *f*_*l*_, between the variable for correlation (${\hat{v}}_{i}\odot {\hat{v}}_{i+l}$) and that for anti-correlation ($-{\hat{v}}_{i}\odot {\hat{v}}_{i+l}$) reflects the non-correlated part of the motions under the constraint of backbones. We emphasize that such a symmetry is present only when the correlation is measured in referring to the backbone coordinates (Fig. 1(a–c)). It displays the *isotropy* of thermal motions of monomers under the constraint of backbones. Quantitatively, the vanishing mean

9 $$\langle {\hat{v}}_{i}\odot {\hat{v}}_{i+l}\rangle \equiv {\int }_{-1}^{1}\,{f}_{l}(x)xdx$$

as *l* → ∞, is a necessary condition for such a symmetry. For those cases considered in Fig. 4, the magnitudes of their means for a pair separated by *l* = 16 bonds are less than 0.01 (filled black circles in Fig. 5(a)). The same computation with the inner product defined in the ordinary way for the first moment $\langle {\hat{v}}_{i}\cdot {\hat{v}}_{i+l}\rangle$ (filled black circles in Fig. 5(b)), by using the simulation-box coordinates, leads to a larger value for each snapshot, than its counterpart $\langle {\hat{v}}_{i}\odot {\hat{v}}_{i+l}\rangle$ (in Fig. 5(a)) in referring to backbone coordinates. The results indicate the unique status of the backbone coordinates in quantifying the correlation properties. The difference between *f*_16_ and the distribution for unconstrained (full) isotropy (abbreviated as ‘UI’), *f*_UI_(*x*) = 0.5, measured by the normed square deviation

10 $${\Vert {f}_{16}-{f}_{{\rm{UI}}}\Vert }^{2}\equiv {\int }_{-1}^{1}\,{({f}_{16}(x)-{f}_{{\rm{UI}}}(x))}^{2}dx,$$

goes monotonically with *s* (filled black circles in Fig. 5(c)), indicating the anisotropy in motion is dominated by chain stiffness.

Note that, the properties of *f*_*l*_’s basically have no direct dependence on the static properties of the conformation of backbone. The statement is indeed true for the decay length *l*_0_ (the persistent length) of the correlation between the tangential backbone directions $\langle {\hat{b}}_{k}\cdot {\hat{b}}_{k+l}\rangle$ at the site-indexed distance *l*, which is an exponentially decaying function in *l*. While the shapes of distributions *f*_*l*_’s are qualitatively dispersed over a wide range among those cases, the persistent lengths are robustly around *l*_0_ ≈ 10. The systems under quasi-steady situations are, therefore, maintaining a kind of dynamic balance, characterized by the ‘parity symmetry’ in *f* between $\hat{v}\cdot \hat{b}$ and −$\hat{v}\cdot \hat{b}$ (Fig. 2(a)) and by the ‘correlation-anti-correlation symmetry’ in *f*_*l*_, for large *l’s*, between ${\hat{v}}_{i}\odot {\hat{v}}_{i+l}$ and −${\hat{v}}_{i}\odot {\hat{v}}_{i+l}$ (Fig. 4). Detailed analysis on the conditional probability of the quantities ${\hat{v}}_{i}\odot {\hat{v}}_{i+l}$ and ${\hat{v}}_{i}\cdot \hat{b}$ reveals that the two escalated wings in the curves of ${f}_{l}({\hat{v}}_{i}\odot {\hat{v}}_{i+l})$ around the end of parallel-bound (${\hat{v}}_{i}\odot {\hat{v}}_{i+l}\approx 1$) and that of antiparallel-bound (${\hat{v}}_{i}\odot {\hat{v}}_{i+l}\approx -1$), are contributed by those motions, of both ${\hat{v}}_{i}$ and ${\hat{v}}_{i+l}$, inclined to be perpendicular to the tangential directions of the backbones, with their events contributing to the centre parts of the curves of $f(\hat{v}\cdot \hat{b})$ in Fig. 2(a).

It is worth to mention at this point that, in all cases above, the distributions of the tangent directions $\hat{b}$ of the backbones are isotropic in space. In systems of chains undergoing clustering, the external isotropy become destroyed during the processes. One important issue is then how the validity of Eq. (4) responds to such a change. In the following, we proceed to probe the issues by considering aggregation processes over the whole time spans of relaxations.

### Symmetry in Conformation and Isotropy of Motion

We consider first a case of aggregation process, over which the system is totally out of the status of quasi-steady situation. The process occurs in a pure system of homopolymer chains^48^, with rigid bonding (i.e. *s* = ∞) and vanishing angle potentials (i.e. *s*_A_ = −∞), in absence of background fluid (*N*_*F*_ = 0). Over the process, the system has *neither* a well-defined velocity distribution specified by Eq. (4) with a virtually fixed *q* for monomers; *nor* has it the symmetry in the distribution *f*_*l*_ for direction cosine ${\hat{v}}_{i}\odot {\hat{v}}_{i+l}$ between a pair of monomers at large separation *l* along a chain. In Fig. 5(a), the first moment of *f*_*l*_, Eq. (9), for *l* = 16 (red diamonds), in snapshots of three arbitrarily picked time spots during the process, which possess correspondingly well- separated values in fitted *q*’s, are much larger than those for the other systems plotted in the figure. The result indicates the motions of the monomers are not in ‘backbone-constrained isotropy’ over the process, during which the backbone tangent orientation $\hat{b}$ is loosing isotropy.

The observation does not, however, imply that a non-equilibrium system with emerging anisotropy in conformation would necessarily loose the symmetry in ${f}_{l}({\hat{v}}_{i}\odot {\hat{v}}_{i+l})$. The aggregation process occurred in a mixture system of chains of rigidly bonded (*s* = ∞) monomers, with the tiny but non-vanishing conformation potentials specified by *s*_A_ = −4 (Fig. 1(e)), in presence of a background fluid^48^, does display the ‘backbone-constrained isotropy’ in motion. It possesses virtually fixed *q* over the process. The data in Fig. 5 (black open circles) display that the first moment of *f*_*l*_ for *l* = 16 is also vanishingly small (Fig. 5(a)), which indicates the presence of ‘backbone-constrained isotropy’ in the system.

We extend the analysis to other systems with *s*_A_ = −4 and consider the cases with *s* = 2, 3 and 4, respectively. These systems were prepared by the same procedure as those considered in Figs 3 and 4, with polymer chains mixed with background fluid. We monitor the effective number of degrees of freedom per monomer,

11 $${\nu }_{eff}\equiv \frac{{{\bf{K}}}_{P\mathrm{,1}}{\nu }_{F}}{{{\bf{K}}}_{F\mathrm{,1}}},$$

as the alternative parameter for the ratio Γ (Eq. (7)), in identifying the reaching of thermal equilibration with the background fluid. We would have the convergent value *ν*_*eff*_ = *ν*_*F*_ = 3 for a collection of non bonded monomers and ${\nu }_{eff}=\frac{2n+1}{n}$ for rigid polymer chains, each composed of *n* monomers.

**Figure 6:**
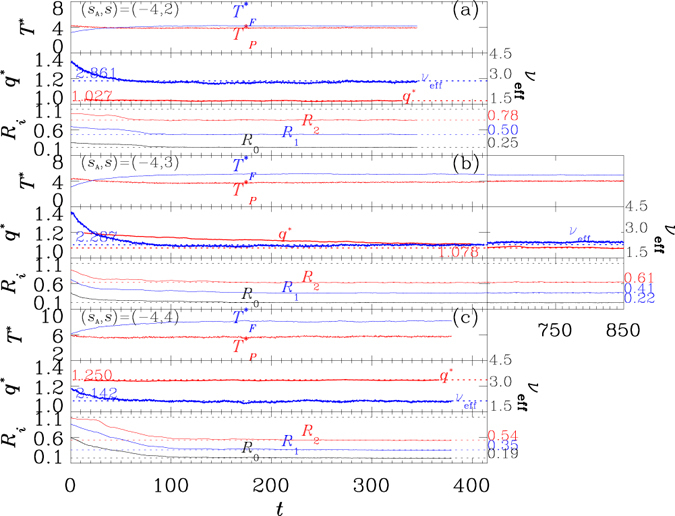
Time evolutions of systems with shared *s*_A_ = −4 and with (**a**) *s* = 2; (**b**) *s* = 3 and (**c**) *s* = 4, respectively. In each case, the top panel shows the instantaneous temperatures ${T}_{P}^{\ast }$ and ${T}_{F}^{\ast }$ for polymer and for background fluid, respectively; the middle panel shows the effective number of degrees of freedom *ν*_eff_ per monomer and the fitted q*; the bottom panel shows the *R*-parameters: *R*_0_, *R*_1_, and *R*_2_. The colored dashed lines in each bottom panel are plotted to show the reaching of steady values for the *R*_0_, *R*_1_ and *R*_2_ parameters, respectively. The systems were prepared as uniform configurations, with *R*_0_, *R*_1_ and *R*_2_ staying close to unity (in *t* < 0, not shown in the plots). The initiation of the growth is signaled by a rapid falling in *R*_0_, corresponding to the entry into stage II on the triggering of paralleled segments in stage I (Fig. 1(d)). It is followed sequentially by the fallings of *R*_1_ and *R*_2_, which mark the reaching of stage III and stage IV, respectively. The latter (stage IV) is featured by the formation of bundled structures (Fig. 7) which may start immediately after the initiation of the previous one (stage III)^48, 49^; the system in plot (c) has an apparent well separated initiations for the two stages. Each data point for q* is an average on 100 data points over a shift window of Δ*t* = 25*τ*, which is 100 times of that used for Figs 2(b) and 5, in computing the values of the parameter. q* and *ν*_eff_ are plotted in spacing of 0.25*τ*. The curves of both quantities are guided by horizontal dashed lines to show their convergence towards constants, except that of q* in plot (b) for the system with *s* = 3. In the latter system, q* decreases with time *t*, in contrast to *ν*_eff_ which barely changes. Data in an extended simulation of the system are also plotted on the right of in plot (b), which shows continuing decreasing trend in q*. The factor *ν*_eff_, on the other hand, encounters a major change separately from that in q*.

**Figure 7:**
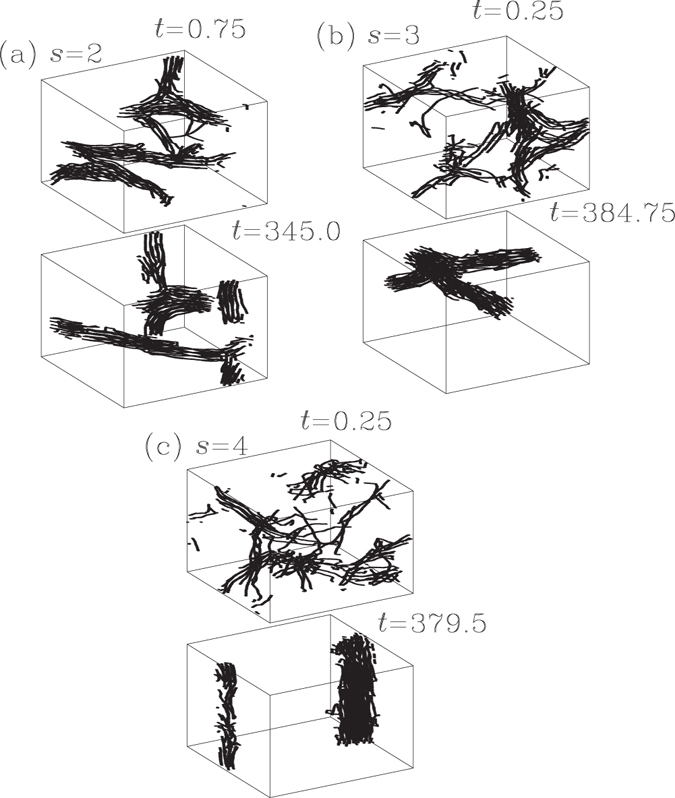
Plots of two typical snapshots of the 40 polymer chains, for each of the three systems with shared *s*_A_ = −4 and with distinctive *s*, (**a**) *s* = 2; (**b**) *s* = 3; (**c**) *s* = 4. The snapshot on the top of each plot is one in approaching to thermal equilibration between polymer and background fluid. The configuration shows that the aggregation of polymer chains proceeds as well. The snapshot on the bottom of each plot displays a steady configuration near the completion of the aggregation. We put, in each plot, the corresponding time spot in Fig. 6 for each of these snapshots.

All of the three systems undergo aggregation processes and the mechanisms are quantified by the set of *R*-parameters (see *Methods*), where the decreasing in the values of *R*_0_, *R*_1_ and *R*_2_^48, 49^ signals the entering of stages II, III and IV, respectively, depicted in Fig. 1(d). Over the processes (Fig. 6, with typical snapshots shown in Fig. 7), the values of q*, averaged over a shifting time window of width Δ*t* = 25*τ*, for the systems with *s* = 2 and 4, respectively, remain constants (middle panels of Fig. 6(a,c) respectively.). The system with *s* = 3, however, has a decreasing q* (middle panel of Fig. 6(b)), despite of the reaching of equilibration between polymer and background fluid signaled by the constancy in *ν*_*eff*_. An extended simulation (on the right of Fig. 6(b)) shows that the adjustment in the effective degrees of freedom of the polymer chains proceeds as well.

In Figs 2(b) and 5(a–c), we include the data for snapshots at some time spots of these three systems, one snapshot for each of the two systems, with *s* = 2 and *s* = 4, respectively (in black open circles) and three snapshots for the system with *s* = 3 (in blue open circles). Over the aggregation processes, the motions of the monomers along the chains in these three systems stay at the status of having ‘backbone-constrained isotropy’, manifested by the presence ‘correlation-anti-correlation symmetry’ in ${f}_{l}({\hat{v}}_{i}\odot {\hat{v}}_{i+l})$, for *l* = 16. The averaged values of ${\hat{v}}_{i}\odot {\hat{v}}_{i+16}$ are all vanishingly small (Fig. 5(a)). While the normed deviation for the snapshots of the systems with *s* = 2 and 4, respectively, are in agreement with the curve determined by the family with *s*_A_ = −1 (filled black circles), those for the three snapshots of the system with *s* = 3 are apparently not on the curve (Fig. 5(c)).

To comprehend the seemingly dispersed situations around the fittings to Eq. (4) among those three systems, which maintain the backbone-constrained isotropy in motions of monomers over the symmetry-breaking non-equilibrium aggregation processes, we go back to examine the implicit assumptions made behind the equations of fittings. Our observations suggest, in the quasi-steady situation under the constraints of backbones, the orientation dependence of velocity is on the angle between $\hat{v}$ and $\hat{b}$, specified by $\hat{v}\cdot \hat{b}$, that is,

12 $$P(v)=\sum _{\hat{v}\cdot \hat{b}}\,{P}_{c}(v|\hat{v}\cdot \hat{b})f(\hat{v}\cdot \hat{b}),$$

where *P*_*c*_ is a conditional probability for *v* under the given $\hat{v}\cdot \hat{b}$ and $f(\hat{v}\cdot \hat{b})$ is the distribution of the variable $\hat{v}\cdot \hat{b}$. The expression Eq. (12) is valid under the condition that the dependence of the joint probability of the three variables *v*, $\hat{v}$ and $\hat{b}$ is independent of the variable $\hat{b}$. The condition considers the backbone coordinates as the relevant variables for statistical quantities and is supported by the unique status of the coordinates to demonstrate symmetry between the correlation and the anti-correlation of monomer motions.

Based on Eq. (12) the data analyzed in Fig. 6 suggest the following scenarios. For uniform distributed $\hat{v}\cdot \hat{b}$, $f(\hat{v}\cdot \hat{b})=0.5$, the variable $\hat{v}\cdot \hat{b}$ has virtually no dominance on the speed variable *v*. The distribution *P*(*v*) is simply the standard Maxwell’s speed distribution for isotropic motions, Eq. (5). Increasing *s* results in the enhanced deviation from the uniform distribution for $f(\hat{v}\cdot \hat{b})$ (Fig. 2). For the two cases with (*s*_A_, *s*) = (−4, 3) and (*s*_A_, *s*) = (−4, 4), respectively, their values of *q* parameter are greater than unity. The large stiffness in the latter system (*s* = 4) seems render the conditional probability ${P}_{1}(v|\hat{v}\cdot \hat{b})$ unaffected by the supposedly enhanced interplays between different chains, in contrast to that for the former system (*s* = 3), where the *q* parameter in Eq. (4) varies with the changes with time. Since the decreasing q* is also observed for the system with (*s*_A_, *s*) = (−1, 3), the chains of which do not aggregate, in extending the time span covered in the previous simulation^51^ to the same orders considered in Fig. 6(c), the tendency should be mainly due to the reaching of time scales sufficiently to allow the softening of the biased modes that had kept the chains staying in the balanced non-equilibrium situations. Such an internal property for individual chains may less sensitive to the external changes in symmetry of conformation encountered over the aggregation process. The data of the extended evolution in Fig. 6(b) suggest that the time span required for the full relaxation in reaching equilibrium for the system with the set of parameters (*s*_A_, *s*) = (−4, 3) would be well beyond the time covered by the current simulation. Such a time of relaxation is even increased for systems with larger *s*.

To sum up the results of our analysis, the implicitly balance maintained dynamically by backbones can be present for chains with non-vanishing angle potentials (*K*_A_ > 0 or ${s}_{{\rm{A}}}\ne -\infty$), which underscores the importance of three-body and four-body interactions (described by the bending angle and torsion angle potentials, respectively). For these systems, the chain stiffness determines the statistical properties of the motions of the monomers under the balance. For chains with weak stiffness (smaller *s*), the thermal motions of monomers follow the standard Maxwell-Boltzmann statistics. In enhancing the chain stiffness (by increasing *s*), the monomers may stay in biased modes with anisotropic motions, with respect to the backbone coordinates, for prolonged time spans before these biased modes could relax to partition the energy to all allowed modes. We reveal from the simulation data novel forms of symmetry and isotropy for monomer motions under the constraints of backbones for systems either possess or never undergo aggregation processes. Our observations may underlie the empirically observed statistical properties for the speed distribution in non-equilibrium.

## Discussion

A thermodynamic quasi-static process involves small changes from one equilibrium state to another, where the displacements at microscopic level can be divided into the contributions producing a macroscopic work *δ* **W** and those generating uncontrolled heat *δ* **Q** (Eq. (1)). The latter are distinguished from the former by displaying spatial isotropy in the motions of particles, supported by the uncorrelated randomness. Such a scenario is not necessarily effective for processes occurring in a system of polymer chains with strong backbone-constraints on the motions of the monomers in a spatially restrictive environment with changes occurring over a time span not allowing the system sufficiently probing over entire phase space. The analysis in this study shows, nevertheless, the using of the major language of equilibrium statistical mechanics to describe those non-equilibrium processes is feasible. The scenario of a ‘quasi-steady’ situation is identified for the system, where the monomer thermal motion characterized by a kind of uncorrelated randomness under the constraints of backbones. Such a property is far beyond triviality in that, the uncorrelated randomness behind the spatial isotropy in thermal motion is identified, in this context, via the correlation-anti-correlation symmetry of directions of motion measured in the backbone coordinates. The symmetry is valid strictly only for directions of motion recorded in the manner referred to the curved backbone coordinates (Fig. 1). The presence of such a property is closely related to the support provided by the three-body and four-body interactions along the backbones, because the system fails to maintain the required implicit stability if the polymer chains have zero angle potentials.

In summary, we have extended the scenario of a ‘quasi-steady’ non-equilibrium situation^51^, to comprise the implicitly balanced situations over aggregation processes. We have shown in our model studies that backbone connectivity guides not only the symmetry for the conformation of the polymer chains but also the allocation of kinetic energy and, thereof, the thermal statistics. Under the governance of backbones in a softly confined environment, the kinetic energy of the monomers may be trapped in the transverse modes and stay for an extended time span before it able to be partitioned to all modes. Over the time span, a kind of stability is implicitly sustained, leading to the correlation-anti-correlation symmetry in the directions of monomer motions and the emerging of a class of generalized Maxwell-Boltzmann statistics. In the strong stiffness limit, the statistics can be described consistently by one single temperature-reduced scaling distribution (Eq. (3)) over the entire simulation time after initial transients. The same is true for the weak stiffness limit, where the scaling distribution resumes the unit-less Gaussi an form (Eq. (2)). In between the two extremes, we can still have scaled distributions prevailing over shorter time spans. The tendency is independent ofwhether, or not, the polymer chains undergo symmetry-breaking aggregation processes.

Figure 3 shows that for systems with strong springs to connect neighboring monomers in polymer chains, the speed distribution of monomers in the parallel (along the direction of the spring to connect two neighboring monomers) and perpendicular directions have different effective temperatures and such systems are in non-equilibrium states. The non-equilibrium behaviors of peptide chains have also been observed in native collagen fibril^69, 70^ and oxyhemoglobin crystals^71^. When one increase the temperature of native collagen fibril, the measured curves for Young’s modulus and logarithmic decrement are very different for different rates to increase the temperature, i.e. the native collagen fibril shows glassy behavior, similar to the spin glass^72^ which has slow relaxation; such slow relaxation at low temperatures can be slower than critical slowing down at the critical point^2, 15^. Taking Fig. 3 and experimental data of native collagen fibril^69, 70^ and oxyhemoglobin crystals^71^ together, one would like to propose that interacting polymer chains under some situations can help biological systems to maintain in non-equilibrium states^2, 15^, even for very long times such as that of ancient date seeds^13, 14^.

In the present study, the total number of monomers and solvent particles is only 10^4^. By using a more powerful computing system and parallel algorithm, Komatsu, *et al*.^73^ had used MD to simulate fluid turbulence with particle number up to 3.779136 × 10^8^. It would be interesting to use computing resources of the order of ref. 73 to simulate polymer-solvent systems with larger numbers of monomers and solvent particles, and with refined structures. One can then study, for example, the influence of polymer chain length and orientation varieties at smaller scales, to underscore their effects on the phase boundary of Fig. 1(e), the speed distribution function of Fig. 3, etc.

The effectiveness of the fitting of velocity distributions by Eq. (4) and, thereof, its temperature-reduced form, Eq. (6) suggests the connection between the stability maintained by the backbones revealed in this study and the *q*-Gaussian based statistical mechanics. Indeed, the *q*-Gaussian statistics has been found for thermal fluctuations in spatially heterogeneous systems^74, 75^, as the generalization of Maxwell-Boltzmann statistics, prevailing in the non-ergodic situations^76^. In some what different context, Brito, *et al*. used *q*-exponential function ${e}_{q}^{x}$, to study the statistics of degree distribution in classes of spatially constructed complex networks, similar to our observations, the value for the parameter *q* has been shown to be continually tuned by some strength-related parameter^77^.

Simulations with enhanced data statistics will reveal more insights on issues, such as how the *q*-Gaussian distribution Eq. (4) of total speed *v*, is related to the distributions of those for the components *v*_‖_, *v*_⊥1_ and *v*_⊥2_. A theoretical construction to produce a *q*-Gaussian analog of Eq. (8) will clarify more precisely why Eq. (4) is still fine in the cases when the components *v*_‖_, *v*_⊥1_ and *v*_⊥2_ have different effective temperatures. Note that there are built-in intrinsic correlations among the components in *q*-Gaussian statistics^67^, in contrast to the scenario behind the elliptical Maxwell-Boltzmann distribution, Eq. (8), where the components are statistically independent and the factor *j*_*E*_(*v*) is purely geometrical. On account of the algebraic property of *q*-exponential that ${x}^{n}{e}_{q}^{x}$ can be decomposed as the sum of (*n* + 1) terms each in form of ${e}_{q^{\prime} }^{ax}$, the factor *j*_*G*_(*v*) in Eq. (4) carries effectively a spectrum of *q*’s in the distribution and is not necessary merely a geometric factor. With intensive simulations of larger size systems, one can explore further the statistical properties of correlations by treating the correlations as variables (Figs 2 and 4). It will be helpful to construct the *q*-generalized statistical relationship between the direction components of velocities and the speed for the anisotropic situations and to refine the knowledge of the statistics behind Fig. 5(c).

## Methods

### Interaction Potential

The total interaction potential (Fig. 1) of a collection of polymer chains is modeled by

13 $$U=\sum _{\langle ij\rangle }^{\prime} \,{V}_{{\rm{VW}},ij}({r}_{ij})+{k}_{{\rm{n}}.{\rm{n}}.}\sum _{\langle ij\rangle }^{\prime\prime} \,{V}_{{\rm{n}}.{\rm{n}}.}({r}_{ij})+{K}_{{\rm{A}}}[\sum _{\langle ijk\rangle }\,{\rm{\Theta }}({\theta }_{ijk})+\sum _{\langle ijkl\rangle }\,{\rm{\Phi }}({\varphi }_{ijkl})].$$

In previous work, we separate strength parameters, denoted by *s*_b_ and *s*_t_, respectively, for the bending angle potential and the torsion angle potential. Since we always assign the same value for the two parameters, we use one single parameter *s*_A_ to unify the notations. The first term is the sum of each Van der Waal potential ${V}_{{\rm{VWij}}}({r}_{ij})$ specific to the inter-chain or non-nearest-neighbor-intra-chain or monomer-fluid interaction sites *i* and *j*, at a distance *r*_*ij*_. This term is the only one has a site-dependent potential ${V}_{{\rm{VW}},ij}$, indexed by *ij*. In the model^51, 52^, Lennard-Jones potential, ${V}_{{\rm{VWij}}}({r}_{ij})=4{\epsilon }_{ij}(({r}_{ij}/{\sigma }_{ij}{)}^{-12}-{({r}_{ij}/{\sigma }_{ij})}^{-6})$, is used. We assign different sets of values for (*σ*_*ij*_, ${\epsilon }_{ij}$) to specify the interaction between monomers, that between fluid atoms and the fluid-monomer interaction, denoted by (*σ*, $\epsilon$), (*σ*_*F*_, ${\epsilon }_{F}$) and (*σ*_*PF*_, ${\epsilon }_{PF}$), respectively. In simulation, we assign ${\epsilon }_{PF}$ = ${\epsilon }_{F}$ = $\epsilon$ and *σ*_*PF*_ = *σ* and ${\sigma }_{F}=\frac{1}{4}\sigma$. In monomer-fluid interaction, however, we drop the attractive part and retain only the *r* ^−12^-potential. A tiny amount (5%) of impurity monomers are introduced, to have the same interactions as those of fluid atoms. These monomers are the only sources of heterogeneity for the polymer chains. The second terms of *U* is the sum of every intra-chain potential between each nearest neighbor pair *i* and *j* along each chain, with *V*_n.n._ and *k*_n.n._, respectively, defining the function form and the strength of the potential, which are common to all such pairs. *V*_n.n._ is realized as a harmonic potential ${V}_{{\rm{n}}.{\rm{n}}.}({r}_{ij})=\frac{1}{2}{k}_{0}{({r}_{ij}-{b}_{ij})}^{2}$ if *k*_n.n._ is finite. Otherwise, in the case of *k*_n.n._ = ∞, a scheme^51^ is applied to generate constraint force keeping *r*_*ij*_ numerically strictly to the value *b*_*ij*_. The ruggedness along the backbone of each chain is specified by the angle potentials in the third and the fourth summations of *U*. They are, respectively, functions of the bending angle *θ*_*ijk*_ determined by three consecutive monomers *i*, *j* and *k* and of the torsion angle ${\varphi }_{ijkl}$ for four consecutive monomers *i*, *j*, *k* and *l*, along a chain. The functions ${\rm{\Theta }}({\theta }_{ijk})={\varepsilon }_{{\rm{b}}}{(\cos {\theta }_{ijk}-\cos {\theta }_{0})}^{2}$ and ${\rm{\Phi }}({\varphi }_{ijkl})={\sum }_{\iota =0}^{3}\,{({\varepsilon }_{{\rm{t}}})}_{\iota }{(\cos {\varphi }_{ijkl})}^{\iota }$ define the functional forms of the potentials. To cover a range of varieties in the major properties of the backbones, revealing their effects on the clustering, we extend the united-atom model of polyethylene (PE), which has a specified value 67.187^o^ for the parameter *θ*_0_ of the potential ${\rm{\Theta }}$ and assign, in our model, the value *θ*_0_ = 112.813^o^. We use unit-less strength parameters ${K}_{{\rm{A}}}={10}^{{s}_{{\rm{A}}}}$ and *k*_n.n._ = 10^*s*^ to cover ranges of magnitudes in powers of ten. All quantities are expressed in terms of the length parameter *σ* and the energy parameter $\epsilon$ of the Lennard-Jones potential between a pair of non-neighboring monomers along a chain (Fig. 1). For model of polyethylene (*K*_A_ = 1, *k*_n.n._ = 1 and the bond length ${\overrightarrow{b}}_{i,i+1}=0.357\,\sigma$), the values for *σ*, $\epsilon$ and *τ* take the values *σ* = 4.3 Å, $\epsilon$ = 57 *K* − *k*_B_ (*k*_B_: Boltzmann constant) and *τ* = 2.2 picoseconds, respectively. The time step of integration used in the simulations for all data shown in this paper is *δt* = 2.5 × 10^−5^ *τ*..

Figure 1(a) shows local coordinates at site *i*, defined by the direction vector ${\hat{b}}_{i}={\overrightarrow{b}}_{i,i+1}/|{\overrightarrow{b}}_{i,i+1}|$ of the bond vector ${\overrightarrow{b}}_{i,i+1}$ from site *i* to site *i* + 1, the unit vector ${\hat{u}}_{i}$ normal to the plane extended by sites *i* − 1, *i* and *i* + 1 and the unit vector ${\hat{w}}_{i}$ = ${\hat{b}}_{i}\times {\hat{u}}_{i}$. ${\theta }_{i-\mathrm{1,}i,i+1}$ is the angle between the bond vectors ${\overrightarrow{b}}_{i-\mathrm{1,}i}$ and ${\overrightarrow{b}}_{i,i+1}$ and ${\varphi }_{i-\mathrm{1,}i,i+\mathrm{1,}i+2}$ is the angle between the normal directions ${\hat{u}}_{i}$ and ${\hat{u}}_{i+1}$ of the two planes. Figure 1(b) shows how to compute the direction cosine in the curved coordinates. The relationship between the direction of motion (unit vector) ${\hat{v}}_{i}\equiv \frac{{\overrightarrow{v}}_{i}}{|{\overrightarrow{v}}_{i}|}$ (red arrow in Fig. 1(a)) of site *i*, with velocity ${\overrightarrow{v}}_{i}$ and the local coordinates is revived at site *i* + *l*, to obtain the unit vector ${({\hat{v}}_{i})}_{i+l}$ (in light red color in Fig. 1(b)). The inner product ${({\hat{v}}_{i})}_{i+l}\cdot {\hat{v}}_{i+l}$ or, equivalently, ${\hat{v}}_{i}\cdot {({\hat{v}}_{i+l})}_{i}$, is denoted by ${\hat{v}}_{i}\odot {\hat{v}}_{i+l}$ in this paper.

### Preparation

With the setup, we can easily control the susceptibility of the system to aggregate, simply by tuning the values of *K*_A_. All systems in this study contain *N*_*P*_ = 40 chains, each composed of *n* = 100 monomers, mixed with a background vapor with *N*_*F*_ = 6000 background spherical atoms, in a cubic simulation box of size *L* = 33.39*σ* on each edge. The persistent length of the chains ranges from 6 to15 times of bond lengths indicating stiff backbones and their end-to-end distance is less than half of *L* and their gyration of radius is around one-tenth of the fully extended chain length. The linear dimension *L* of the simulation box is not much larger than these characteristic lengths. Under such a circumstance, it is difficult to justify the assumption made for equilibrium statistical mechanics without controversy. The latter applies to the situation that the size of individual molecules be much smaller than the dimension of the whole system. Such a ‘softly confined’ allocation in simulation, on the other hand, reflects certain features encountered in confined biological environments.

Note that the analysis carried out in Fig. 3 takes data from very short periods because of temperature variations for simulated systems under no thermal controls, in contrast to those in Figs 2(a) and 4, where the quantities are derived from direction vectors. The latter vectors carry no direct dependence on temperature and a time period of 125 times of that in Fig. 3 to take averages for each curve. The intervals for Fig. 2(a) are (chosen arbitrarily) from the quasi-steady situations starting at *t* = 29.4 (for *s* = 0), *t* = 42.15 (for *s* = 1), *t* = 41.9 (for *s* = 2), *t* = 41.65 (for *s* = 3), *t* = 59.65 (for *s* = 4) and *t* = 169.2 (for *s* = ∞) respectively, of Fig. 4 in ref. 52.

### R-parameters

We define the parameter^48, 49^

14 $${R}_{k}=\equiv \frac{{\int }_{0}^{\tfrac{L}{2}}\,{g}_{{\rm{inter}}}(r){r}^{k}dr}{{\int }_{0}^{\tfrac{L}{2}}\,{\rm{\Xi }}({g}_{{\rm{inter}}}(r)){r}^{k}dr},$$

for *i* = 0, 1 and 2, where ${\rm{\Xi }}(x)=1$ if and only $x\ne 1$. The decreasing in the values of *R*_0_, *R*_1_ and *R*_2_^48, 49^ signal the entering of stages II, III and IV, respectively (in Fig. 1(d)). Stage II specifies the longitudinal growth to have ribbon-like local structures. The orderings in stages III and IV are in transverse directions to form belt-like (two-dimensional) and bundle-like (three-dimensional) structures, respectively. The initiation of the aggregation process is signaled by a rapid falling in *R*_0_, corresponding to the triggering of parallelized segments. It is followed by the growths in transverse directions. Since the systems under consideration in this study are dilute in polymer concentration, it is insufficient to form extended planar crystal structures^50^ and, instead, the chains grow into bundles^48, 49^ (Fig. 7). It is these parameters that are used to determine the schematic diagram of steady phases in Fig. 1(e).

### Notations

We list the notations for interaction potentials, parameters and statistical quantities in Tables 1, 2 and 3, respectively.

**Table 1:** List of Interaction Potentials.

${\sum }_{\langle ij\rangle }^{^{\prime} }{V}_{{\rm{VW}},ij}({r}_{ij})$	summation of all inter-chain and non-nearest-neighboring pairs of monomers and fluid-fluid and fluid-monomer pairs
*V*_VW,*ij*_(*r*_*ij*_)	van der Waal interaction between site *i* and site *j*, at a distance *r*_*ij*_
${\sum }_{\langle ij\rangle }^{^{\prime\prime} }{V}_{{\rm{n}}{\rm{.n}}.}({r}_{ij})$	summation over all nearest-neighbor pairs along polymer chains
*V*_n.n._(*r*_*ij*_)	harmonic potential for finite *k*_n.n._
${\sum }_{\langle ijk\rangle }\,{\rm{\Theta }}({\theta }_{ijk})$	summation over all consecutive monomers, indexed by *i*, *j* and *k* along backbones with a bending angle *θ*_*ijk*_
${\rm{\Theta }}({\theta }_{ijk})$	bending angle potential
${\sum }_{\langle ijkl\rangle }\,{\rm{\Phi }}({\varphi }_{ijkl})$	summation over all consecutive monomers, indexed by *i*, *j*, *k* and *l* along backbones with a torsion angle ${\varphi }_{ijkl}$
${\rm{\Phi }}({\varphi }_{ijkl})$	torsion angle potential

**Table 2:** Parameters.

*k*_n.n._	strength parameter for bonds between a pair of nearest-neighbor along a chain
*s*	*k*_n.n._ = 10^*s*^
*K*_A_	strength parameter for angle potentials
*s*_A_	${K}_{{\rm{A}}}={10}^{{s}_{{\rm{A}}}}$
*σ*	length parameter
$\epsilon$	energy parameter
*τ*	time parameter
*N*_*P*_	number of polymer chains in the system
*n*	number of monomers in a polymer chain
*N*_*F*_	number of fluid atoms in the system
*q*, *β*	parameters in generalized Maxwell-Boltzmann distribution for single snapshot
q*, Δ*q*	mean-value and mean-square-deviation of fitted values of *q* over a time interval
*R*_0_	parameter to identify longitudinal growth along backbones
*R*_1_	parameter to identify transverse growth to form belt-like structure
*R*_2_	parameter to identify transverse growth to form bundle-like structure
*ν*_*P*_	number of degrees of freedom per monomer
*ν*	number of degrees of freedom
*ν*_*F*_	number of degrees of freedom per fluid atom
*ν*_*eff*_	effective number of degrees of freedom per monomer
Γ	parameter for identifying the reaching of thermal equilibration between polymer and fluid

**Table 3:** Statistical Quantitties.

*P*(*v*)	probability density function of speed
*P*_GMB_(*v*)	generalized Maxwell-Boltzmann probability density function of speed
*P*_EMB_(*v*)	elliptic Maxwell-Boltzmann probability density function of speed
$f(\hat{v}\cdot \hat{b})$	probability density function of the inner product between directions of monomer motion $\hat{v}$ and the bond $\hat{b}$
${\hat{v}}_{i}\cdot {\hat{v}}_{i+l}$	inner product along a chain between direction vectors ${\hat{v}}_{i}$ of site *i* and ${\hat{v}}_{i+l}$ of site *i* + *l* computed in simulation-box coordinates
${\hat{v}}_{i}\odot {\hat{v}}_{i+l}$	inner product along a chain between direction vectors ${\hat{v}}_{i}$ of site *i* and ${\hat{v}}_{i+l}$ of site *i* + *l* computed in backbone coordinates
${f}_{l}({\hat{v}}_{i}\odot {\hat{v}}_{i+l})$	probability density function of the inner product ${\hat{v}}_{i}\odot {\hat{v}}_{i+l}$

## References

[1] Stanley, H. E. *Introduction to Phase Transitions and Critical Phenomena* (Oxford Univ. Press, New York, 1971).

[2] Hu C-K (2014). Historical Review on Analytic, Monte Carlo, and Renormalization Group Approaches to Critical Phenomena of Some Lattice Models. Chin. J. Phys..

[3] Yang CN, Lee TD (1952). Statistical theory of equations of state and phase transitions. I. Theory of condensation. Phys. Rev..

[4] Lee TD, Yang CN (1952). Statistical theory of equations of state and phase transitions. II. Lattice gas and Ising model. Phys. Rev..

[5] Blöte HWJ, Luijten E, Heringa JR (1995). Ising universality in three dimensions: a Monte Carlo study. J. Phys. A: Math. Gen..

[6] Talapov AL, Blöte HWJ (1996). The magnetization of the 3D Ising model. J. Phys. A: Math. Gen..

[7] Sengers JV, Shanks JG (2009). Experimental Critical-Exponent Values for Fluids. J. Stat. Phys..

[8] Watanabe H, Ito N, Hu C-K (2012). Phase diagram and universality of the Lennard-Jones gas-liquid system. J. Chem. Phys..

[9] Okumura H, Okamoto Y (2004). Liquid-gas phase transitions studied by multibaric-multithermal Monte Carlo simulations. J. Phys. Soc. Jpn..

[10] Onuchic JN, Wolynes PG (2004). Theory of protein folding. Curr. Opin. Struc. Bio..

[11] Dobson CM (2003). Protein folding and misfolding. Nature.

[12] Hu C-K (2015). Proteins aggregation and human diseases. J. Phys.: Conference Series.

[13] Sallon S (2008). Germination, genetics, and growth of an ancient date seed. Science.

[14] Yashina S (2012). Regeneration of whole fertile plants from 30,000-y-old fruit tissue buried in Siberian permafrost. Proc. Natl. Acad. Sci. USA.

[15] Hu C-K (2013). Slow dynamics in proteins and polymer chains. AIP Conf. Proc..

[16] Orr WJC (1947). Statistical treatment of polymer solutions at infinite dilution. Trans. Faraday Soc..

[17] Vogel T, Bachmann M, Janke W (2007). Freezing and collapse of flexible polymers on regular lattices in three dimensions. Phys. Rev. E.

[18] Lee JH, Kim S-Y, Lee J (2012). Exact partition function zeros of a polymer on a simple cubic lattice. Phys. Rev. E.

[19] Chen C-N, Hsieh Y-H, Hu C-K (2013). Heat capacity decomposition by partition function zeros for interacting self-avoiding walks. EPL.

[20] Hsieh Y-H, Chen C-N, Hu C-K (2016). Exact partition functions of interacting self-avoiding walks on lattice. EPJ Web of Conf..

[21] Hsieh Y-H, Chen C-N, Hu C-K (2016). Efficient algorithm for computing exact partition functions of lattice polymer models. Comp. Phys. Comm..

[22] Li MS (2010). Factors Governing Fibrillogenesis of Polypeptide Chains Revealed by Lattice Models. Phys. Rev. Lett..

[23] Co NT, Hu C-K, Li MS (2013). Dual effect of crowders on fibrillation kinetics of polypeptide chains revealed by lattice models. J. Chem. Phys..

[24] Schreck JS, Yuan J-M (2010). Exactly solvable model for helix-coil-sheet transitions in protein systems. Phys. Rev. E.

[25] Zamparo M, Trovato A, Maritan A (2010). Simplified Exactly Solvable Model for beta-Amyloid Aggregation. Phys. Rev. Lett..

[26] Xiao X, Wu M-C (2014). Simplified lattice model for polypeptide fibrillar transitions. Phys. Rev. E.

[27] Izmailian NS, Wu M-C, Hu C-K (2016). Finite-size corrections and scaling for the dimer model on the checkerboard lattice. Phys. Rev. E.

[28] Khandogin J, Brooks CL (2007). Linking folding with aggregation in Alzheimer’s beta-amyloid peptides. Proc. Nat. Aad. Sci. USA.

[29] Itoh SG, Okamoto Y (2008). Amyloid-*β* (29–42) dimer formations studied by a multicanonical-multioverlap molecular dynamics simulation. J. Phys. Chem. B.

[30] Rojas A, Liwo A, Browne D, Scheraga HA (2010). Mechanism of fiber assembly; treatment of A*β*-peptide aggregation with a coarse-grained united-residue force field. J. Mol. Biol..

[31] Nguyen PH, Tarus B, Derreumaux P (2014). Familial Azheimer A2 V Mutation Reduces. the Intrinsic Disorder and Completely Changes the Free Energy Landscape of A*β*1-28 Monomer. J. Phys. Chem. B.

[32] Okumura H, Itoh SG (2014). Amyloid fibril disruption by ultrasonic cavitation: nonequilibrium molecular dynamics simulations. J. Am. Chem. Soc..

[33] Knowles TPJ, Buehler MJ (2011). Nanomechanics of functional and pathological amyloid materials. Nature Nanotech..

[34] Petkova AT (2002). A structural model for Alzhei fibrils based on experimental from solid state NMR. Proc. Nat. Aad. Sci. USA.

[35] Keten S, Xu Z, Ihle B, Buehler MJ (2010). Nanoconfinement controls stiffness, strength and mechanical toughness of *β*-sheet crystals in silk. Nature Mater..

[36] Wu MC (2011). Universal geometrical factor of protein conformations as a consequence of energy minimization. EPL.

[37] Banavar JR (2007). Structural motifs of biomolecules. Proc. Natl. Acad. Sci. USA.

[38] Nguyen HD, Hall CK (2004). Molecular dynamics simulations of spontaneous fibril formation by random-coil peptides. Proc. Natl. Acad. Sci. USA.

[39] Pellarin R, Caflisch A (2006). Interpreting the Aggregation Kinetics of Amyloid Peptides. J. Mol. Bio..

[40] Urbanic B, Cruz L, Yun S, Buldyrev SV, Bitan G, Teplow DB, Stanley HE (2004). Discrete Molecular Dynamics Simulations of Peptide Aggregation. Proc. Natl. Acad. Sci. USA.

[41] Doye JPK, Frenkel D (1998). Mechanism of Thickness Determination in Polymer Crystals. Phys. Rev. Lett..

[42] Lu PJ (2008). Gelation of particles with short-range attraction. Nature.

[43] Saw S, Ellegaard NL, Kob W, Sastry S (2011). Computer simulation study of the phase behavior and structural relaxation in a gel former modeled by three-body interactions. J. Chem. Phys..

[44] So M (2011). Ultrasonication-Dependent Acceleration of Amyloid Fibril Formation. J. Mol. Bio..

[45] Baldwin AJ (2011). Metastability of Native Proteins and the Phenomenon of Amyloid Formation. J. Am. Chem. Soc..

[46] Yang JX, Gould H, Klein W (1988). Molecular-dynamics investigation of deeply quenched liquids. Phys. Rev. Lett..

[47] Ma WJ, Banavar JR, Koplik J (1992). A molecular dynamics study of freezing in a confined geometry. J. Chem. Phys..

[48] Ma WJ, Hu CK (2010). Molecular Dynamics Approach to Aggregation of Polymer Chains with Monomers Connected by Rigid Bonds. J. Phys. Soc. Jpn..

[49] Ma WJ, Hu CK (2010). Molecular Dynamics Approach to Aggregation of Polymer Chains with Monomers Connected by Springs. J. Phys. Soc. Jpn..

[50] Yamamoto T (2013). Molecular dynamics of polymer crystallization revisited: Crystallization from the melt and the glass in longer polyethylene. J Chem Phys.

[51] Ma WJ, Hu CK (2010). Generalized Statistical Mechanics and Scaling Behavior for Non-equilibrium Polymer Chains: I. Monomers Connected by Rigid Bonds. J. Phys. Soc. Jpn..

[52] Ma WJ, Hu CK (2010). Generalized Statistical Mechanics and Scaling Behavior for Non-equilibrium Polymer Chains: II. Monomers Connected by Springs. J. Phys. Soc. Jpn..

[53] Andersen HC (1980). Molecular dynamics simulations at constant pressure and/or temperature. J. Chem. Phys..

[54] Tuckman, M. E. *Statistical Mechanics*: *Theory and Molecular Simulation* (Oxford University Press, New York, 2010).

[55] Nose S (1984). A unified formulation of constant-temperature molecular dynamics methods. J. Chem. Phys..

[56] Okumura H, Okamoto Y (2004). Molecular dynamics simulations in the multibaric-multithermal ensemble. Chem. Phys. Lett..

[57] Parrinello M, Rahman A (1980). Crystal structure and pair potentials: a molecular dynamics study. Phys. Rev. Lett..

[58] Parrinello M, Rahman A (1981). Polymorphic transitions in single crystals: a new molecular dynamics method. J. Appl. Phys..

[59] Eisenmenger F, Hansmann UHE, Hayryan S, Hu CK (2001). [SMMP] A modern package for simulation of proteins. Comp. Phys. Comm..

[60] Eisenmenger F, Hansmann UHE, Hayryan S, Hu CK (2006). An enhanced version of SMMP-open-source software package for simulation of proteins. Comp. Phys. Comm..

[61] Tsallis C (1988). Possible generalization of Boltzmann-Gibbs statistics. J. Stat. Phys..

[62] Tsallis, C. In *Complexity and Nonextensivity*: *New Trends in Statistical Mechancis*, edited by Abe, S., Sakagami, M. & Suzuki, N. *Proceedings of International Workshop*, *Prog*. *Theor*. *Phys*. *Suppl*. **162** (2006).

[63] Anderson, T. W. *An introduction to multivariate statistical analysis*, 3rd. ed. (Wiley, New York, 2003).

[64] Doi, M. & Edwards, S. F. *The theory of polymer dynamics* (Oxford University Press, New York, 1988).

[65] Banavar JR, Cieplak M, Maritan A (2004). Lattice tube model of proteins. Phys. Rev. Lett..

[66] Perkins TT, Smith DE, Chu S (1994). Direct observation of tube-like motion of a single polymer chain. Science.

[67] Silva R, Plastino AR, Lima JAS (1998). A Maxwellian path to the q-nonextensive velocity distribution function. Phys. Lett..

[68] Onoratoa M, Vozellaa L, Promentb D, Lvov YV (2015). Route to thermalization in the *alpha*-Fermi-Pasta-Ulam system. Proc. Nat. Aad. Sci..

[69] Gevorkian SG, Allahverdyan AE, Gevorgyan DS, Hu CK (2011). Glassy state of native collagen fibril?. EPL.

[70] Gevorkian SG (2013). Stabilization and anomalous hydration of collagen fibril under heating. Plos One.

[71] Gevorkian SG, Allahverdyan AE, Gevorgyan DS, Hu C-K (2015). Thermal-induced force release in oxyhemoglobin. Sci. Rep..

[72] Dasgupta C, Ma SK, Hu CK (1979). Dynamic properties of a spin glass model at low temperatures. Phys. Rev. B.

[73] Komatsu TS, Matsumoto S, Shimada T, Ito N (2014). A glimpse of fluid turbulence from the molecular scale. Int. J. Mod. Phys. C.

[74] Combe G, Richefeu V, Stasiak M (2015). Experimental Validation of a Nonextensive Scaling Law in Confined Granular Media. Phys. Rev. Lett..

[75] Livadiotis G, McComas DJ (2009). Beyond kappa distributions: Exploiting Tsallis statistical mechanics in space plasmas. J. Geophys. Res.: Space Phys..

[76] Tirnakli U, Borges EP (2016). The standard map: From Boltzmann-Gibbs statistics to Tsallis statistics. Sci. Rep..

[77] Brito S, da Silva LR, Tsallis C (2016). Role of dimensionality in complex networks. Sci. Rep..

